# Improvement of Learning and Memory Induced by* Cordyceps* Polypeptide Treatment and the Underlying Mechanism

**DOI:** 10.1155/2018/9419264

**Published:** 2018-03-15

**Authors:** Guangxin Yuan, Liping An, Yunpeng Sun, Guangyu Xu, Peige Du

**Affiliations:** College of Pharmacy, Beihua University, Jilin 132013, China

## Abstract

Our previous research revealed that* Cordyceps militaris* can improve the learning and memory, and although the main active ingredient should be its polypeptide complexes, the underlying mechanism of its activity remains poorly understood. In this study, we explored the mechanisms by which* Cordyceps militaris* improves learning and memory in a mouse model. Mice were given scopolamine hydrobromide intraperitoneally to establish a mouse model of learning and memory impairment. The effects of* Cordyceps* polypeptide in this model were tested using the Morris water maze test; serum superoxide dismutase activity; serum malondialdehyde levels; activities of acetyl cholinesterase, Na+-k+-ATPase, and nitric oxide synthase; and gamma aminobutyric acid and glutamate contents in brain tissue. Moreover, differentially expressed genes and the related cellular signaling pathways were screened using an mRNA expression profile chip. The results showed that the genes* Pik3r5*,* Il-1β*, and* Slc18a2* were involved in the effects of* Cordyceps* polypeptide on the nervous system of these mice. Our findings suggest that* Cordyceps* polypeptide may improve learning and memory in the scopolamine-induced mouse model of learning and memory impairment by scavenging oxygen free radicals, preventing oxidative damage, and protecting the nervous system.

## 1. Introduction

Learning and memory are among the main functions of the human brain, and together they play an important role in biological evolution and development [1]. Learning and memory are higher physiological activities in the brain and also the core components of cognitive function [2]. With increasing age, declining learning and memory abilities represent a common phenomenon and most of the population has been affected by hypomnesia [3]. Moreover, cognitive deficits develop during the progression of various neurological diseases, including Alzheimer's disease (AD) and Parkinson syndrome (PD) [4]. Therefore the early prevention and treatment of these symptoms of declining learning and memory have attracted much attention, in part due to their very broad potential market [5].

At present, the drugs commonly used clinically for prevention and improvement of learning and memory impairment include free radical scavengers, drugs to prevent beta amyloidal deposits formation, M receptor agonists, and acetyl cholinesterase inhibitors. Unfortunately, these few types of drugs used to treat learning and memory impairment commonly present poor effects regarding learning memory, toxicity, adverse side effects, and no preventive effect against the occurrence and progression of neurological diseases. Traditional Chinese medicines have shown some advantages over the traditional drugs for diseases involving learning and memory impairment [6–8], such as fewer and less severe side effects, more action targets, and low cost. Thus, there is much interest in identifying effective drugs among traditional Chinese medicines for the prevention and improvement of impaired learning and memory.* Cordyceps militaris* (North* Cordyceps sinensis*) is a rarely used Chinese herbal medicine at present, with bioactivities such as improvement of immunity [9–11], bacteriostasis [12, 13], and antihypertension [14]. Previous studies by our research group demonstrated that* Cordyceps militaris* can improve the learning and memory, and its main active ingredient is likely its polypeptide complexes [15–17].

To investigate the mechanism responsible for this effect of* Cordyceps militaris* on learning and memory, in the present study, we first established a mouse model of acquired learning and memory impairment via intraperitoneal injection of scopolamine hydrobromide. Using this model, we examined the effects of* Cordyceps* polypeptide on the learning and memory abilities of the mice by observing mouse behavior serum superoxide dismutase (SOD) activity; serum malondialdehyde (MDA) content; the acetyl cholinesterase AChE, Na^+^-k^+^-ATPase, and endothelial nitric oxide synthase (eNOS) activities; and the gamma aminobutyric acid (GABA) and glutamate (Glu) contents in mouse brain tissues. Furthermore, mice were treated with* Cordyceps* polypeptides prepared via an enzymolysis approach, and an mRNA expression microarray was used to screen the differentially expressed genes and related signaling pathways in the brain tissues of treated mice. Overall, the ability of* Cordyceps* polypeptide to prevent and improve learning and memory impairment in the model mice was confirmed, and the data obtained for the related mechanisms may provide an important theoretical basis for the further research and development of* Cordyceps* polypeptide.

## 2. Experimental Materials and Methods

### 2.1. Experimental Materials

#### 2.1.1. Animals

A total of 120 eight-week-old male ICR mice (specific-pathogen free grade), weighing 18~22 g, were purchased from Liaoning Changsheng Biotechnology Co., Ltd. (license number: SCXK (Liao)-2015-0001). The mice were housed individually in an environment of controlled temperature (20 ± 1°C) and humidity (40–70%) and subjected to 12 h light/dark cycle with free access to food and water. The study was conducted according to the European Community Guidelines for the Use of Experimental Animals, and the Ethics Committee of Beihua University approved the study protocol.

#### 2.1.2. *Cordyceps militaris*

Fruiting bodies of* Cordyceps militaris* were purchased from Shenyang Nizi* Cordyceps* Cultivation Base.

#### 2.1.3. Reagents and Instruments

Piracetam Tablets (Northeast Pharmaceutical Group Shenyang First Pharmaceutical Co. Ltd.; batch number: 5141224); scopolamine hydrobromide (Chengdu Manchester Stewart Biological Technology Co. Ltd., batch number: 150417) and pepsin (activity: 3000~3500 u/mg; Beijing Dingguo Changsheng Biotechnology Co., Ltd.); acetylcholinesterase (AChE), *γ*-aminobutyric acid (GABA), Na+-K+-ATPase, glutamic acid (Glu), monoamine oxidase-B (MAO-B), and endothelial nitric oxide synthase (eNOS) assay kits (Shanghai Shangle Biological Products Research Institute, batch number: 30126078); malondialdehyde (MDA) and superoxide dismutase (SOD) assay kits (Nanjing Jiancheng Biological Products Research Institute, batch number: THZ-C); a constant temperature oscillator (Taicang Experimental Equipment Factory); an Infinite M200 ELIASA (TECAN), 5430R low-temperature high-speed centrifuge (Eppendorf Company, USA), an AL204 electronic balance (Mettler-Toledo Instruments Co. Ltd.), and a Morris water maze apparatus (Chengdu Taimeng Technology Co. Ltd.) were used in this study.

### 2.2. Animal Experiments

#### 2.2.1. Preparation of* Cordyceps* Polypeptide [18]

An appropriate amount of the dried fruiting body of* Cordyceps militaris* was ground into powders (40 mesh). The powder was then defatted with petroleum ether and the residue was dried. After drying, 50 g of the dried residue was dissolved in 450 mL distilled water and the solution was adjusted to pH 2.0 with 1 M HCl. Then 10 g pepsin was added to the solution at 37°C for enzymolysis for 5 h. After the enzymolysis, the solution was heated at 100°C in a water bath for 10 min for inactivation of the pepsin. Next it was filtered and subjected to centrifugation for collection of the supernatant and was lyophilized to obtain a* Cordyceps* polypeptide powder (5.36 g).

#### 2.2.2. Animal Grouping and Administration

One hundred twenty mice were randomly divided into six groups according to treatment: a blank control group, the model group, a positive control (drug) group, a high-dose* Cordyceps* polypeptide group (900 mg·kg^−1^), a middle-dose* Cordyceps* polypeptide group (450 mg·kg^−1^), and a low-dose* Cordyceps* polypeptide group (225 mg·kg^−1^). Mice in the positive control group were given the positive control drug Piracetam Tablets at a dose of 600 mg·kg^−1^ (calculated based on body surface area), and mice in the blank control and model groups received the same volume of distilled water intragastrically once daily for 45 consecutive days.

#### 2.2.3. Animal Model Preparation and Animal Training

Prior to the behavioral testing, the mice were placed in the water maze apparatus and allowed to swim freely for 2 min on 2 consecutive days, for adaptation of the mice to the environment. One week before the intragastric administration of drug, the water maze test began. At 1 h after the intragastric administration, the mice in the blank control group were given an intraperitoneal injection of saline (3 mL·kg^−1^) and mice in the other groups received an intraperitoneal injection of scopolamine hydrobromide (3 mg·kg^−1^) for the establishment of an acquired mouse learning and memory impairment model, and 15 min later the Morris water maze test was carried out for 7 consecutive days.

#### 2.2.4. Morris Water Maze Test

The Morris water maze apparatus consisted of two parts, a cylindrical tube and recording device. The cylindrical tube (diameter 80 cm, height 30 cm) was divided into four quadrants (quadrants 1, 2, 3, and 4). A platform with a diameter of 5 cm was placed at the center of quadrant 3. Before testing, the tube was filled with water up to 11 cm below the rim. The water temperature was maintained at 20 ± 2°C, and an appropriate amount of titanium dioxide was added to the water to make it ivory. The platform was located at 1-2 cm below the surface of the water. When the test was carried out, the surrounding environment was kept unchanged.

*The Positioning Navigation Test*. The test was performed on days 1–6. Before the start of the test, the mice were allowed to stay on the platform for 30 s. Then the mice were placed into the water facing the pool wall in any two quadrants except the quadrant containing the platform. Escape latencies within 120 s were recorded and average escape latency values were calculated as the evaluation indexes.

On day 7, the spatial search test was carried out. For this text, the platform was removed, and then the mice were placed into the water in a fixed quadrant, facing the wall of the pool. The number of errors made by the mice while crossing the place where the platform had been within 120 s was recorded, and the average values were calculated as the evaluation indexes.

#### 2.2.5. Animal Sampling and Biochemical Index Detection Method

Following the Morris water maze test, the eyeballs of the mice were removed with clean forceps for the collection of 1 mL of whole blood. The blood samples were centrifuged at 3000 r/min at 4°C for 10 min to obtain the serum, and the serum samples were stored at −20°C until use. The whole brain tissues of mice were harvested, weighed, and prepared into a 10% homogenate upon addition of saline at a ratio of 1 : 9 (w : v) in an ice water bath. The homogenate was centrifuged at 3500 r/min at 4°C for 10 min to obtain the supernatant, and the supernatant was stored at −80°C until use. In accordance with the instructions for the detection kits, the serum SOD activity and MDA content were measured, the AChE, Na^+^-k^+^-ATP, and eNOS activities were assessed, and the GABA and Glu contents in the brain tissues were determined according to the instructions for the enzyme-linked immunosorbent assay kits.

### 2.3. RNA Extraction and Quality Control

The total liver RNA was extracted with Trizol reagent (Invitrogen, Gaithersburg, MD, USA) by following the operating instructions. The liver tissue was ground into powders under the condition of liquid nitrogen freezing, and the powders were transferred into a 1.5 mL centrifuge tube in which Trizol (500 mg liver tissue per 0.5 mL Trizol) had been preadded before the liquid nitrogen completely volatilized. The liver tissue-Trizol homogenate was drawn strongly several times with a 5 mL disposable syringe until the homogenate was no longer sticky, in order to fully break down the cells and genomic DNA, which was kept at room temperature for use. Chloroform was added to the homogenate in a ratio of 200 *μ*L chloroform per 1 mL Trizol used, which was strongly oscillated for 30 s to be mixed evenly, kept at room temperature for 5 min, and then centrifuged at 12000 rpm for 15 min to obtain the supernatant. The supernatant was carefully transferred to another centrifuge tube, and more attention should be paid to drawing the supernatant to avoid taking the mesophase. The supernatant was added with 500 uL isopropanol to be precipitated at room temperature for 10 min; the solution was centrifuged at 12000 rpm for 15 min and the supernatant was discarded; the residue was added with 1 mL of 75% ethanol to wash the RNA, which was oscillated by vortex oscillation, kept at room temperature for 5 min, and then centrifuged at 7500 rpm for 5 min; the supernatant was discarded, and the residue was added with 1 mL of 75% ethanol to be washed once again. The residue-ethanol solution was centrifuged at 7500 rpm for 5 min, the supernatant was thrown away, and the residue was dried at room temperature. Please note that the RNA precipitation should not be too dry; otherwise, it will not be dissolved easily.

The RNA was dissolved in an appropriate amount of TE buffer (10 mmol/L Tris, pH7.6, 1 mmol/L EDTA). The total RNA mass was detected by agarose gel electrophoresis and the mRNA was purified in accordance with the instructions of RNeasy Mini Kit (Qiagen, Valencia, CA, USA). According to the kit instructions, the quality of RNA samples was assessed, and the integrity of RNA samples, inhibitors, and DNA pollution were detected. The quality of mRNA was detected by RNA formaldehyde-agarose gel electrophoresis, and the content of RNA was determined by UV spectrophotometry. The same amount of RNA taken from each mouse in each group was mixed and then used for the chip detection.

### 2.4. Gene Chip Analysis

#### 2.4.1. Sample Labeling and Hybridization

Before RNA labeling, the Agilent ND-1000 was used to detect RNA degradation and determine RNA concentration. For gene chip analysis, samples were labeled with the Agilent Quick Amp Labeling kit and hybridized with Agilent SureHyb.

Sample labeling and array hybridization were performed according to the Agilent One-Color Microarray-Based Gene Expression Analysis protocol (Agilent Technology). Briefly, total RNA from each sample was linearly amplified and labeled with Cy3-UTP. The labeled cRNAs were purified by RNeasy Mini Kit (Qiagen). The concentration and specific activity of the labeled cRNAs (pmol Cy3/*μ*g cRNA) were measured by NanoDrop ND-1000. 1 *μ*g of each labeled cRNA was fragmented by adding 11 *μ*l 10x Blocking Agent and 2.2 *μ*l of 25x fragmentation buffer and then heated at 60°C for 30 min, and finally 55 *μ*L 2x GE hybridization buffer was added to dilute the labeled cRNA. 100 *μ*L of hybridization solution was dispensed into the gasket slide and assembled to the gene expression microarray slide. The slides were incubated for 17 hours at 65°C in an Agilent Hybridization Oven. The hybridized arrays were washed, fixed, and scanned using the Agilent DNA Microarray Scanner (part number G2505C).

#### 2.4.2. Data Acquisition and Standardization

After washing, the chip was scanned with an Agilent DNA Microarray Scanner. The Agilent Feature Extraction software (v11.0.0.1) was used to acquire chip probe signal values, and the Agilent Gene Spring GX v12.1 software was used for standardization of the chip results. Points located outside of the 95% confidence interval represented differentially expressed genes.

#### 2.4.3. Significance Analysis of the Functions of Differentially Expressed Genes

Gene ontology (GO) annotation of the differentially expressed genes was performed using the NCBI Gene Ontology database to identify all the GO categories in which the genes were involved, and Fisher exact test and the *X*^2^ test were applied to calculate the significance level and error rate for each GO. The error rate was then used to calibrate the *P* values, consequently screening out the significant GO categories reflected by the differentially expressed genes (*P* < 0.05) [19]. The experimental results were analyzed using European Bioinformatics Institute (EBI) database [20].

#### 2.4.4. Functional and Biological Pathway Enrichment Analyses of Differentially Expressed Genes Identified by mRNA Expression Chip Using DAVID 

From the open DAVID database (https://david.ncifcrf.gov/), 450 genes were submitted within the gene sets for further analysis, and at the same time, the corresponding gene identifier (gene identifier corresponding to the gene name OFFICIAL_GENE_SYMBOL) was selected. The complete genome of the mouse was selected as including the background genes, and then the “Functional Annotation Tool” was applied as the analysis tool, so that the results of the GO enrichment analysis and pathway enrichment analysis of differentially expressed genes could be obtained [21].

### 2.5. Real-Time Quantitative Polymerase Chain Reaction (PCR) Verification

Real-time quantitative PCR validation of the differentially expression of specific genes associated with the immunomodulation achieved by* Cordyceps* polypeptide in mice was conducted in strict accordance with the instructions for the Real-time PCR Master Mix (SYBR Green, Toyobo) [22]. The instrument used was the Roche Light Cycler 1.5, and the primer sequences are provided in Table 1.

### 2.6. Statistical Analysis

Statistical analyses were performed using SPSS 16.0 statistical software (SPSS, Inc.). The single factor analysis of variance and *q* test of the measurement data were performed with the analysis of variance (ANOVA) program, and least squared differences were used for the pairwise comparison. The ranked data were analyzed by Rid it. *P* < 0.05 was considered statistically significant [23].

## 3. Experimental Results

### 3.1. Effects of* Cordyceps* Polypeptide on Behavior of Mice with Learning and Memory Impairment

In the positioning navigation test on days 1–6, the escape latency during the first 3 days did not differ among mice in the different groups, but on days 4–6, the escape latency of mice in the model group was significantly prolonged compared with that of mice in the control group (*P* < 0.01), indicating that the model was successfully established. On days 4 and 5, the escape latencies of mice in both the high-dose* Cordyceps* polypeptide group and the positive drug group were also significantly shortened compared with that of mice in the model group (*P* < 0.05). By day 6, the escape latencies of mice in all* Cordyceps* polypeptide-treated groups and the positive drug group were significantly shortened compared with that of mice in the model group (*P* < 0.05). In the spatial search test on day 7, the number of times that the mice crossed the platform in the model group was significantly less than that in the control group (*P* < 0.05), whereas the numbers of platform crossings in the high-dose* Cordyceps* polypeptide group and the positive drug group were significantly greater than that in the control group (*P* < 0.05; Table 2).

### 3.2. Effects of* Cordyceps* Polypeptide on the Serum SOD Activity and MDA Content

Compared with those in the blank control group, the serum SOD activity in the serum of mice was significantly reduced and the MDA content was significantly increased in mice of the model group (*P* < 0.05), further confirming that the model was successfully established. Compared with those in the model group, the serum SOD activity was significantly increased, and the MDA content was significantly reduced in the high-dose* Cordyceps* polypeptide group and the positive control group (*P* < 0.05; Table 3).

### 3.3. Effects of* Cordyceps* Polypeptide on AChE, Na^+^-k^+^-ATPase, and eNOS Activities and GABA and Glu Contents in the Brain Tissue of Mice

The AChE activity was significantly higher (*P* < 0.05), the Na^+^-k^+^-ATPase and eNOS activities were significantly lower (*P* < 0.05), and the GABA and Glu contents were significantly lower (*P* < 0.05) in the brain tissue of mice in the model group compared with those in the blank control group, also confirming that the model was successfully established. Compared with those in the model group, the AChE activities were significantly decreased (*P* < 0.05), the Na^+^-k^+^-ATPase and eNOS activities were significantly increased, and GABA and Glu contents were significantly increased (*P* < 0.05) in the brain tissue of mice in the high-dose* Cordyceps* polypeptide group and the positive control group. However, among these indexes, only the Na^+^-k^+^-ATPase activity was significantly increased in the brain tissue of mice in low-dose* Cordyceps* polypeptide group compared with that in the model group (*P* < 0.05). The corresponding data are presented in Table 4.

### 3.4. mRNA Chip Results

**Figure 1:**
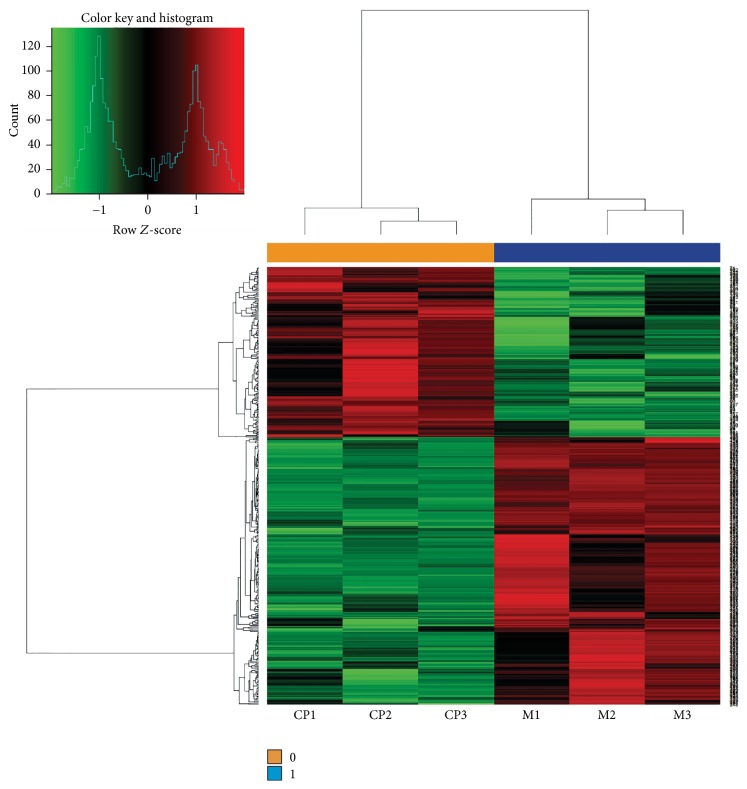
Results of mRNA expression profile microarray analysis (M: model group; CP:* Cordyceps* polypeptide group).

As shown in Figure 1, compared with the model group, there were 450 differentially expressed genes in the brain tissue of mice in the* Cordyceps* polypeptide-treated group, of which the expression of 175 genes (38.89%) was significantly upregulated and that of 275 gene (61.11%) was significantly downregulated. The differences in the mRNA chip results were significant between the* Cordyceps* polypeptide-treated group and the model group, and the results showed that* Cordyceps* polypeptide could significantly inhibit the expression level of genes related to the nervous system (the expression of 61.11% genes was downregulated).

### 3.5. Clustering Results

The GO cluster analysis of the 450 differentially expressed genes was carried out using the DAVID database, and 36 related pathways were found. Seven of these pathways were directly related to the immune function. Statistical analysis for the genes related to these seven pathways showed that multiple genes were involved in more than one of the seven pathways, and further analysis revealed that the pathways involving three genes (PIK3R5, IL-1B, and SLC18A2) were the most (Table 5).

### 3.6. Real-Time Quantitative PCR Verification

**Figure 2:**
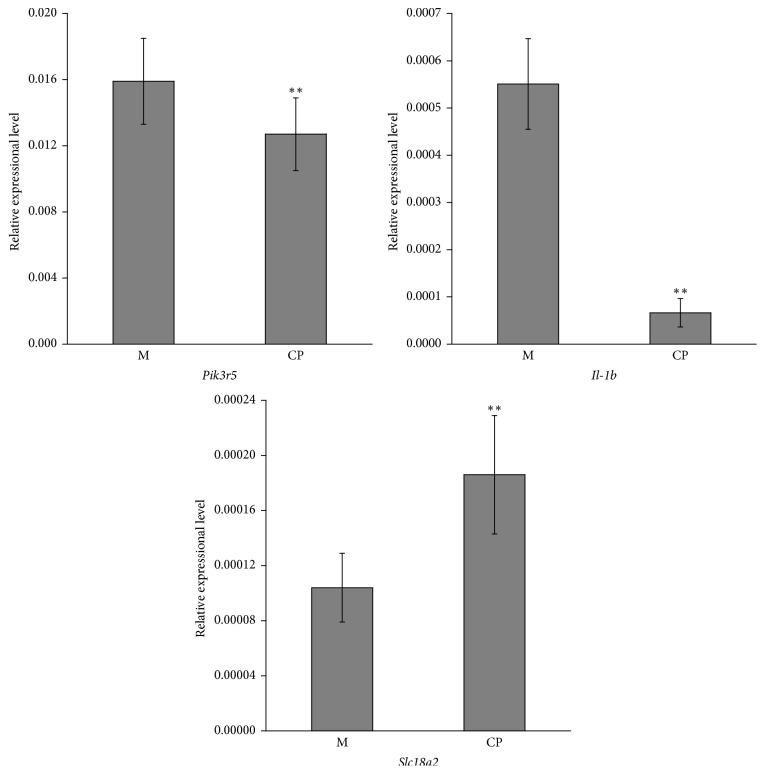
Real-time quantitative PCR validation of* Pik3r5*,* Il-1b*, and* Slc18a2* gene (M: model group; CP:* Cordyceps* polypeptide group; ^*∗∗*^*P* < 0.01).

Real-time quantitative PCR validation of the expression of three genes, including* Pik3r5*,* Il-1β*, and* Slc18a2*, was conducted. As shown in Table 6, the* Pik3r5* and* Il-1β* expression levels were found to be significantly altered in the brain tissue of mice in the model group (*P* = 0.015997, *P* = 0.001177) with expression ratios of 0.8 and 0.12, respectively, between the* Cordyceps* polypeptide-treated group and model group (Figure 2), and this resulted in negative regulation on immune activity. The* Slc18a2* gene was significantly upregulated in the brain tissue of mice with learning and memory impairment (*P* = 0.002095 with an expression ratio of 1.79 between the* Cordyceps* polypeptide-treated group and model group).

## 4. Discussions

With increases in the social pressure and the incidence of various diseases, more and more people, including not only the elderly but also teens, have begun to experience the symptoms of a decline in memory. Therefore, there is a need for drugs or active components that can improve learning and memory. Studies have shown that* Cordyceps militaris* enhances immunity combat aging and promotes cardiovascular regulation, but there have been few reports on the effects of* Cordyceps* polypeptide on learning and memory to our knowledge. In the present study, scopolamine hydrobromide was used to establish a mouse model of learning and memory impairment, and the effect of* Cordyceps* polypeptide on the learning and memory in these mice was investigated from two perspectives, that is, the behavioral one in the Morris water maze test and the molecular detection of related biochemical indexes. The Morris water maze testing showed that* Cordyceps polypeptide* could shorten the latency for finding the platform and increase the number of crossings at the place where the platform was previously placed, indicating that* Cordyceps* polypeptide could improve the learning and memory ability of mice with learning and memory impairment induced by scopolamine.

SOD is involved in the scavenging of oxygen free radicals in the body and thereby prevents lipid peroxidation and protects the cell membrane from oxidative damaged [24]. MDA is one of the products of lipid peroxidation induced upon injury and can aggravate the damage to the cell membrane. Therefore, measurement of the SOD activity and MDA content can be used to evaluate the antioxidant effects. AChE is a neurotransmitter that is closely associated with the function of learning and memory, and an increase in AChE content can damage the cholinergic nerve, causing a cognitive impairment, which means AChE can be used as an indicator for revaluating the learning and memory [25]. GABA and Glu are the central inhibitory and stimulatory neurotransmitters, respectively, and a balance in their expression plays an important role in the maintenance of normal functions of the central nervous system. Na^+^-k^+^-ATPase, also known as sodium potassium pump, is located in the cell membrane and involved in the energy supply. Moreover it can increase cerebral blood flow, promote brain energy metabolism, and improve learning and memory functions. eNOS catalyzes the in vivo synthesis of NO with a protective effect on the nervous system [26]. Promoting the activity of eNOS can increase the release of NO to protect nerve cells. Our results showed that* Cordyceps* polypeptide could improve the serum SOD activity, decrease the MDA content and AChE activity in the mouse brain, increase Na^+^-k^+^-ATPase and eNOS activity, and increase GABA and Glu expression, suggesting that* Cordyceps* polypeptide could improve the capacity for learning and memory in mice with a learning and memory disorder.

In the present study, through mRNA expression microarray analysis, a total of 450 differentially expressed genes were identified between the* Cordyceps* polypeptide-treated group and the model group. Then 36 related pathways were revealed through the clustering analysis for gene function, and the pathways involving three genes* Pik3r5*,* Il-1β*, and* Slc18a2* existed were the most. The real-time quantitative PCR analysis of the expression of* Pik3r5*,* Il-1β*, and* Slc18a2* further indicated that these three genes were all the key factors in the effects of* Cordyceps* polypeptide on the nervous system in the mouse model of learning and memory impairment.

*Il-1β* is a cytokine secreted by T helper 1 (Th1) cells to promote inflammation. Studies have shown that* Il-1β* may be involved in the pathophysiological process of depression, and the response to antidepressant treatment involves reducing the function of the 5-HT system, activating the hypothalamic-pituitary-adrenal (HPA) axis, and affecting the regeneration of neurons [27]. Additional reports have demonstrated that childhood trauma may increase the level of cytokines in vivo and influence the course of depression and the therapeutic effect of antidepressive drugs. Our mRNA chip analysis in this study confirms that* Cordyceps* polypeptide can inhibit the expression of a gene, which may be advantageous for the regeneration of neurons and play a protective role in the nervous system, whereby* Cordyceps* polypeptide may improve the mice's learning and memory of mice [28].

The* Slc18a2* gene is located at chromosomal region 10q25.3 and encodes vesicular monoamine transporter 2 (VMAT2) which can transport dopamine. It also encodes epinephrine and serotonin which cross the membrane into synaptic vesicles and take part in the monoamine metabolism. It has been reported that the physiological roles of these monoamines are associated with motor control, mood stability, and autonomic functions. Normal functioning of this process is essential to maintain dopamine metabolism in substantia nigra neurons.

*Slc18a2* gene expression was reported to reduce the dyskinesia and depression-like behavior in mouse models [29].* Slc18a2* gene expression was also shown to promote the secretion of neurotransmitters when the gene chip technology was applied to observe the effect of arsenic on the expression profile of genes related to the neurotransmitters in the cerebellum of mice. In the present study,* Cordyceps* polypeptide treatment could promote the expression of* Slc18a2* and thereby promoted the secretion of neurotransmitters in mice to reduce dyskinesia and depression-like behavior, which may be conducive to the improvement of learning and memory.

The phosphatidylinositol-3-kinase (PI3K)/Akt and mammalian target of rapamycin (mTOR) pathway, one of the most important intracellular signal transduction pathways, plays important roles in the processes of cell growth, survival, proliferation, and apoptosis as well as in angiogenesis [30]. PI3K family members are important kinases of inositol and phosphatidylinositol as well as important signal transduction molecules, involved in the regulation of cell proliferation, apoptosis, and differentiation process [31]. PIK3R5, a member of the PI3K family, has been rarely studied, and there has been no study linking it to the nervous system [32]. In the present study, real-time quantitative PCR demonstrated significant downregulation of* Pik3r5* (*P* = 0.0002353) with* Cordyceps* polypeptide treatment, and the experimental results of it were more significant than those of the other 2 genes. The downregulation expression in mRNA chip indicates that* Cordyceps* polypeptide may inhibit the expression of the gene, which can be used as a potential drug target of* Cordyceps militaris* for the further experiment research.

## 5. Conclusions

A mouse model of learning and memory impairment was established via injection of scopolamine hydrobromide and the effects of different doses of* Cordyceps* polypeptide delivered intragastrically were investigated by the evaluation of mouse behavior via Morris water maze testing, the measurement of several biological indexes in the serum and brain tissue of the mice, and the identification of differentially expressed genes and the related cellular signaling pathways. The results showed that* Cordyceps* polypeptide could improve the learning and memory in these mice, increase the serum SOD activity, decrease the serum MDA content, reduce AChE activity, increase Na^+^-k^+^-ATPase and eNOS activity, and increase GABA and Glu levels in the brain tissue of the mice. The mRNA expression file chip analysis, clustering analysis of the gene function according to the DAVID database, and real-time qPCR verification indicate that the improvement in learning and memory induced by* Cordyceps* polypeptide treatment in the mouse model of learning and memory impairments is likely related to its effects on* Pik3r5*,* Il-1β*,* and Slc18a2*, and these three genes may be the potential targets for* Cordyceps militaris*.

## Figures and Tables

**Table 1:** Primer pairs for the real-time quantitative PCR.

Gene name	Two-way primer sequence	Annealing temperature (°C)	Product size (bp)
*Pik3r5*	F: 5′ CGGCTTCTATTACTTCAACTTCCA3′ R: 5′ CGGAGGAAGACTTGATAAACAGAC3′	60	71
*Il-1β*	F: 5′ CTTCAGGCAGGCAGTATCACTC3′ R: 5′ GCAGTTGTCTAATGGGAACGTC3′	60	194
*Slc18a2*	F: 5′ TCACCAACCCATTCATAGGACT3′ R: 5′ ATGAGCAAGAGGAGCCGATT3′	60	168

**Table 2:** Morris water maze test results.

Group	Escape latency (s)			Crossing the platform (times)
	4th day	5th day	6th day	7th day
CON	38.12 ± 34.65	33.26 ± 22.16	30.17 ± 22.80	4.20 ± 3.65
M	85.56 ± 43.75^*∗∗*^	74.33 ± 50.61^*∗∗*^	71.14 ± 50.13^*∗∗*^	1.54 ± 1.63^*∗*^
L-CP	74.55 ± 49.26	52.62 ± 49.60	51.07 ± 44.34^#^	2.54 ± 2.47
M-CP	78.87 ± 48.13	54.87 ± 43.41	49.98 ± 35.91^#^	1.93 ± 2.02
H-CP	59.86 ± 51.48^#^	47.30 ± 47.55^#^	39.61 ± 39.12^#^	3.25 ± 1.86^#^
PC	58.23 ± 40.16^#^	48.64 ± 39.73^#^	38.14 ± 26.66^#^	2.64 ± 3.22^#^

^*∗*^ *P* < 0.05 versus blank control group, ^*∗∗*^*P* < 0.05 versus blank control group, ^#^*P* < 0.05 versus model group; CON: blank control group; M: model group; L-CP: low-dose CP group; M-PC: middle-dose CP group; H-CP: high-dose CP group; PC: positive control group.

**Table 3:** Effects of *Cordyceps* polypeptide on the serum SOD activity and MDA content in mice.

Group	SOD (U/mL)	MDA (nmol/mL)
CON	128.72 ± 17.13	5.15 ± 0.91
M	105.19 ± 12.38^*∗*^	6.48 ± 0.86^*∗*^
L-CP	116.73 ± 13.16	5.57 ± 1.08
M-CP	118.20 ± 25.22	5.66 ± 0.89
H-CP	127.57 ± 18.35^#^	4.99 ± 1.51^#^
PC	132.44 ± 16.48^#^	5.16 ± 0.94^#^

^*∗*^ *P* < 0.05 versus blank control group, ^#^*P* < 0.05 versus model group; CON: blank control group; M: model group; L-CP: low-dose CP group; M-PC: middle-dose CP group; H-CP: high-dose CP group; PC: positive control group.

**Table 4:** Effects of *Cordyceps* polypeptide on AChE, Na^+^-k^+^-ATPase, and eNOS activities and GABA and Glu contents in brain tissue of mice.

Group	AchE (ng/mL)	Na^+^-k^+^-ATP (ng/mL)	eNOS (U/mL)	GABA (ng/mL)	Glu (nmol/mL)
CON	15.41 ± 0.71	16.42 ± 1.65	4.62 ± 0.50	85.73 ± 5.87	60.05 ± 3.71
M	16.63 ± 0.87^*∗*^	13.87 ± 2.11^*∗*^	4.04 ± 0.45^*∗*^	76.33 ± 8.80^*∗*^	4.77 ± 5.14^*∗*^
L-CP	15.64 ± 0.94	16.96 ± 2.18^#^	4.60 ± 0.55	84.61 ± 4.46^#^	58.25 ± 3.63
M-CP	15.59 ± 0.75	16.11 ± 2.83	4.62 ± 0.63	84.60 ± 7.99	58.13 ± 4.93
H-CP	15.43 ± 0.90^#^	16.77 ± 1.81^#^	4.65 ± 0.44^#^	87.98 ± 6.45^#^	60.71 ± 1.09^#^
PC	15.35 ± 0.56^#^	17.34 ± 3.17^#^	4.70 ± 0.66^#^	85.24 ± 4.99^#^	60.98 ± 4.34^#^

^*∗*^ *P* < 0.05 versus blank control group, ^#^*P* < 0.05 versus model group; CON: blank control group; M: model group; L-CP: low-dose CP group; M-PC: middle-dose CP group; H-CP: high-dose CP group; PC: positive control group.

**Table 5:** Screened out representative genes.

No.	Genes name	Pathway count	Pathway name
1	*Pik3r5*	4	Chagas disease (American trypanosomiasis) natural toxoplasmosis Leukocyte transendothelial migration Dopaminergic synapse
2	*Il-1β*	3	Chagas disease (American trypanosomiasis) natural toxoplasmosis Prion diseases
3	*Slc18a2*	3	Chagas disease (American trypanosomiasis) Leukocyte transendothelial migration Dopaminergic synapse

**Table 6:** Experimental results of real-time quantitative PCR.

Data comparison scheme	*Pik3r5*	*Il-1b*	*Slc18a2*
M	1.59*E* − 02	5.51*E* − 04	1.04*E* − 04
CP	1.27*E* − 02	6.62*E* − 05	1.86*E* − 04
CP/M	0.80	0.12	1.79
*P* value	0.0002353	0.001177	0.002095

M: model group; CP: *Cordyceps* polypeptide group.
